# A Population-Based Analysis of *BRCA1*/*2* Genes and Associated Breast and Ovarian Cancer Risk in Korean Patients: A Multicenter Cohort Study

**DOI:** 10.3390/cancers13092192

**Published:** 2021-05-02

**Authors:** Kyung-Sun Park, Woochang Lee, Moon-Woo Seong, Sun-Young Kong, Kyung-A Lee, Jung-Sook Ha, Eun-Hae Cho, Sung-Hee Han, Inho Park, Jong-Won Kim

**Affiliations:** 1 Department of Laboratory Medicine, Kyung Hee University School of Medicine and Kyung Hee University Medical Center, Seoul 02447, Korea; drparkkyungsun@khu.ac.kr; 2 Department of Laboratory Medicine, Asan Medical Center, University of Ulsan College of Medicine, Seoul 05505, Korea; wlee1@amc.seoul.kr or; 3 Department of Laboratory Medicine, Seoul National University Hospital, Seoul National University College of Medicine, Seoul 03080, Korea; MWSeong@snu.ac.kr or; 4 Cancer Research Institute, Seoul National University College of Medicine, Seoul 03080, Korea; 5 Division of Translational Science, Research Institute, National Cancer Center, Goyang 10408, Korea; ksy@ncc.re.kr; 6 Department of Laboratory Medicine, Hospital, National Cancer Center, Goyang 10408, Korea; 7 Department of Laboratory Medicine, Yonsei University College of Medicine, Seoul 06273, Korea; KAL1119@yuhs.ac; 8 Department of Laboratory Medicine, Keimyung University School of Medicine, Daegu 41931, Korea; ksksmom@naver.com or; 9 Genomic Research Center, GC Genome, Yongin 16924, Korea; ehcho@gccorp.com; 10 Biotechnology Division, BioCore Co. Ltd., Yongin 16954, Korea; hansungh@bio-core.com; 11 Department of Pathology, Gangnam Severance Hospital, Yonsei University College of Medicine, Seoul 06273, Korea; ihpark20@yuhs.ac; 12 Center for Precision Medicine, Gangnam Severance Hospital, Yonsei University College of Medicine, Seoul 06273, Korea; 13 Department of Laboratory Medicine and Genetics, Samsung Medical Center, Sungkyunkwan University School of Medicine, Seoul 06351, Korea

**Keywords:** *BRCA1*, *BRCA2*, breast cancer, ovarian cancer, variants of uncertain significance, cumulative risk

## Abstract

**Simple Summary:**

Although it has been suggested that cancer risk and genetic variation vary by population, there is still a lack of research on non-European populations. In this study, we applied Korean patients as a model to find out the way to conduct *BRCA1*/*2*-related clinical studies in non-European populations who do not have as much clinical data as Europeans. The *BRCA1/2* variants were classified following the 2015 ACMG standards/guidelines and using a multifactorial probability-based approach. To estimate the additional sample numbers needed to resolve *BRCA1/2* unclassified status, we applied a simulation analysis considering population-specific clinical characteristics. In addition, we estimated the risks of breast or ovarian cancer for *BRCA1*/*2* carriers by mutation regions. Data from this study reveal that *BRCA1*/2 variants in the non-European population are highly specific; therefore, population-specific study is essential for clinical application of treatment or prevention for breast or ovarian cancer.

**Abstract:**

In this study, we performed a comprehensive analysis of *BRCA1*/*2* variants and associated cancer risk in Korean patients considering two aspects: variants of uncertain significance (VUS) and pathogenic or likely pathogenic variants (PLPVs) in *BRCA1* and *BRCA2*. This study included 5433 Korean participants who were tested for *BRCA1/2* genes. The *BRCA1/2* variants were classified following the standards/guidelines for interpretation of genetic variants and using a multifactorial probability-based approach. In Korea, 15.8% of participants had *BRCA1* or *BRCA2* PLPVs. To estimate the additional sample numbers needed to resolve unclassified status, we applied a simulation analysis. The simulation study for VUS showed that the smaller the number of samples, the more the posterior probability was affected by the prior probability; in addition, more samples for *BRCA2* VUS than those of *BRCA1* VUS were required to resolve the unclassified status, and the presence of clinical information associated with their VUS was an important factor. The cumulative lifetime breast cancer risk was 59.1% (95% CI: 44.1–73.6%) for *BRCA1* and 58.3% (95% CI: 43.2–73.0%) for *BRCA2* carriers. The cumulative lifetime ovarian cancer risk was estimated to be 36.9% (95% CI: 23.4–53.9%) for *BRCA1* and 14.9% (95% CI: 7.4–28.5%) for *BRCA2* carriers.

## 1. Introduction

Familial breast–ovarian cancer associated with the *BRCA1* (phenotype MIM #604370) or *BRCA2* (MIM #612555) is an autosomal dominant disorder that has been exceptionally well-studied in terms of diagnosis, treatment, management, and prevention. Especially as Poly (ADP-ribose) polymerase inhibitors have shown clinical benefit for treatment of breast or ovarian cancer with the presence of a *BRCA1* or *BRCA2* pathogenic variant [1,2], identifying these variants is more important than ever.

There are two approaches to classifying *BRCA1* or *BRCA2* genetic variants; one is based on the 2015 American College of Medical Genetics and Genomics (ACMG) and the Association for Molecular Pathology (AMP) standards and guidelines (briefly, 2015 ACMG/AMP) [3], and the other is based on a Bayesian framework set forth by the International Agency for Research on Cancer (IARC) recommendations [4,5]. Recently, the ClinGen Sequence Variant Interpretation Working Group showed that the 2015 ACMG/AMP criteria are compatible with Bayesian statistical reasoning [6]. To date, multiple databases for *BRCA1* or *BRCA2* genetic variants have been developed [7,8,9]. However, most of the data are based on European populations. Although it has been suggested that cancer risk and genetic variation vary by populations [10,11,12], there is still a lack of research on non-European populations.

When conducting *BRCA1-* or *BRCA2*-related studies in non-European populations who do not currently have as much clinical data as Europeans, it is important to predict how many samples are needed to resolve the inconclusive state of variants of uncertain significance (VUS). In addition, as it has been suggested that there are distinct differences in clinical characteristics of breast or ovarian cancer [13,14,15] and in the spectrum of *BRCA1/2* variants between non-Europeans and Europeans [10,11,16,17], questions arise about whether the cancer risk by pathogenic or likely pathogenic variants (PLPVs) in *BRCA1/2* estimated based on Europeans can be used in non-European populations. Here, we performed a comprehensive analysis for *BRCA1*/*2* variants and associated cancer risk in Korean patients with respect to VUS and PLPVs in *BRCA1* and *BRCA2*.

## 2. Results

### 2.1. Participant Characteristics

The Korea ONCOgene Research and Diagnosis (KONCORD) consortium was formed for the purposes of this study. The KONCORD consortium consists of eight major clinical genetics laboratories at university hospitals and commercial genetic laboratories in Korea. The data from KONCORD and a previous cohort [18] were collected. There were 5433 participants enrolled in this study. Demographics of study participants are described in Table S1. In our study cohort, the mean age of diagnosis was 43.5 years (range 19–82, 95% CI: 43.1–43.8 years), and 33.5% of participants had a family history of cancer. Patients with unilateral breast cancer (58.6%) were most frequently enrolled, followed by patients with ovarian cancer (7.4%), bilateral breast cancer (6.4%), and both breast and ovarian cancer (1.3%). The pathologic subtypes of breast or ovarian cancer enrolled are described in Table S2.

### 2.2. Classification of Genetic Variants in BRCA1 or BRCA2 Genes Following 2015 ACMG/AMP and IARC Criteria

**Figure 1:**
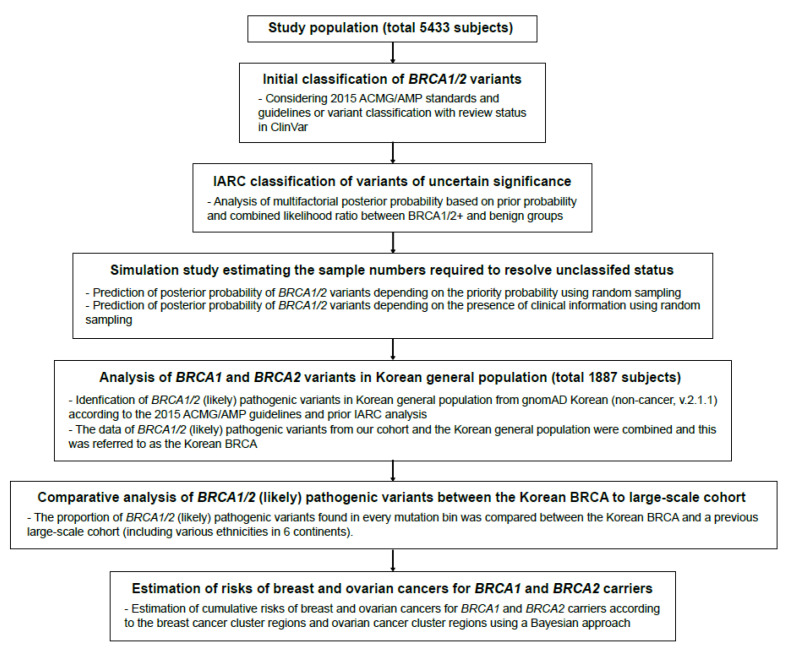
Analysis workflow in this study. A comprehensive analysis of *BRCA1*/*2* variants and associated cancer risk in Korean patients, considering two aspects: variants of uncertain significance (VUS) and pathogenic or likely pathogenic variants (PLPVs) in *BRCA1* and *BRCA2.* The ClinVar (https://www.ncbi.nlm.nih.gov/clinvar/) was accessed on 17 March 2020 and gnomAD (https://gnomad.broadinstitute.org/) was accessed on 13 March 2020. 2015 ACMG/AMP, 2015 American College of Medical Genetics and Genomics (ACMG) and the Association for Molecular Pathology (AMP); BRCA1/2+, carriers of *BRCA1* or *BRCA2* (likely) pathogenic variants; IARC, International Agency for Research on Cancer; P/LP, pathogenic or likely pathogenic variants.

We present the entire analysis workflow in Figure 1. Of the 5433 cases, 15.1% (95% CI: 14.1–16.2%) of participants had *BRCA1* or *BRCA2* PLPVs in the initial analysis; the 125 PLPVs in *BRCA1* were detected in 459 participants (8.4%, 95% CI: 7.7–9.3%), the 111 PLPVs in *BRCA2* were detected in 361 participants (6.6%, 95% CI: 6.0–7.4%), and 2 participants had both *BRCA1* and *BRCA2* PLPVs (both c.5030_5033del (p.Thr1677Ilefs*2) in *BRCA1* and c.1399A>T (p.Lys467*) in *BRCA2* were detected in one bilateral breast cancer patient, and both c.5496_5506delinsA (p.Val1833Serfs*7) in *BRCA1* and c.7480C>T (p.Arg2494*) in *BRCA2* were detected in another bilateral breast cancer patient) (Tables S3 and S4).

For the combined likelihood ratio (LR), we compared personal/family cancer history between the pathogenic (349 cases in BRCA1+, 291 cases in BRCA2+) and benign (2931 cases) groups (Table S5). For pathologic profiles, the BRCA1+ group had a significantly increased likelihood of basal-like subtype breast cancer grade 2 (LR = 4.7, 95% CI: 3.0–7.2) or 3 (LR = 4.6, 95% CI: 3.9–5.5) or high-grade ovarian cancer (LR = 3.4, 95% CI: 2.6–7.5), while the BRCA2+ group had a significantly increased likelihood of luminal subtype breast cancer grades 2 (LR = 1.2, 95% CI: 1.02–1.4) and 3 (LR = 1.5, 95% CI: 1.1–1.9).

Of 44 different VUS in *BRCA1*, 16 (36.4%) were not submitted to ClinVar (accessed March 2020), while 26 (24.8%) of 105 *BRCA2* VUS were not (Tables S6 and S7). In addition, of the *BRCA1* VUS, 39% were not located in functional domains, 37% were located in BRCA1 C Terminus domain (BRCT), 22% in Ring finger domain, and 2% in Serine-rich domain associated with BRCT (by Pfam (http://pfam.xfam.org/ [accessed on 18 April 2021])). Of the *BRCA2* VUS, only 27% were located in functional domains, 10% in the BRCA2 repeat region, 7% in BRCA2 helical, 5% in oligonucleotide/oligosaccharide-binding, domain 1 (OB1), and 2% in oligonucleotide/oligosaccharide-binding, domain 3 (OB3).

The IARC classification of VUS in *BRCA1* or *BRCA2* was analyzed using multifactorial probability. Of 44 different VUS in *BRCA1*, 9 variants were IARC class 1, 16 variants were class 2, 14 variants were class 3, one variant was class 4, and four variants were class 5. Analysis of the 105 different VUS in *BRCA2* indicated that 11 were class 1, 76 were class 2, and 18 were class 3. When we considered the rationale by a previous study that posterior probabilities for any variants with a combined LR between 0.5 and 2 were not calculated [19], the combined LR of any VUS in *BRCA1* was not included in the range, and the combined LR of 58 out of 105 VUS in *BRCA2* were included. Considering the rationale by a previous study [19], the posterior probabilities of only 47 VUS in *BRCA2* could be calculated: there were 11 class 1 VUS, 28 class 2 VUS, and 8 class 3 VUS.

Finally, considering the recommendations by the previous study [20], 15.8% (858 out of 5433, 95% CI: 14.8–16.9%) of participants had *BRCA1* or *BRCA2* PLPVs in our cohort; the 130 PLPVs in *BRCA1* were detected in 495 participants (9.1%, 95% CI: 8.3–10.0%), the 111 PLPVs in *BRCA2* were detected in 361 participants (6.6%, 95% CI: 6.0–7.4%), and 2 participants had both *BRCA1* and *BRCA2* PLPVs.

### 2.3. Simulation Study on the Estimation of Additional Sample Numbers for Resolving Unclassified Status

**Figure 2:**
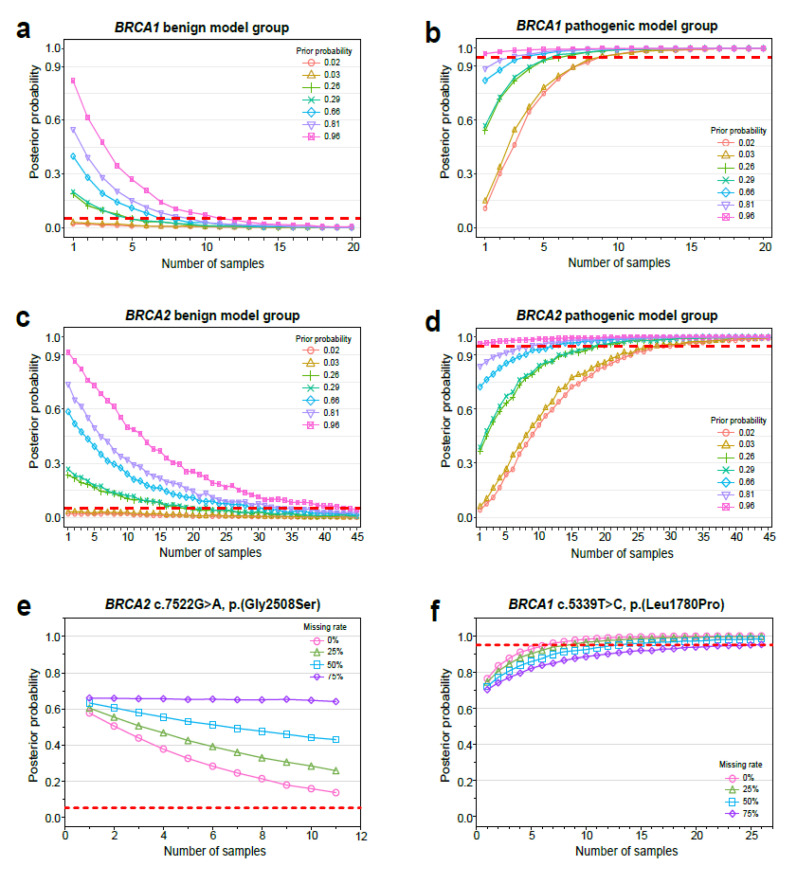
Simulation study estimating the sample numbers required to resolve unclassified status. We created two benign and pathogenic datasets, independent from the benign and pathogenic model groups. The benign set contained samples that had only (likely) benign variants according to the 2015 ACMG/AMP or IARC 1/2 variants, while the pathologic set contained samples that had (likely) pathogenic variants or IARC 4/5 variants. A simulation study on the posterior probability in (**a**) *BRCA1* benign model group, (**b**) *BRCA1* pathogenic model group, (**c**) *BRCA2* benign model group, and (**d**) *BRCA2* benign model group. Simulation study on the posterior probability depending on the presence of clinical information (**e**) at c.7522G>A, p.(Gly2508Ser) in *BRCA2* and (**f**) c.5339T>C, p.(Leu1780Pro) in *BRCA1*. The prior probability of these two variants was the same at 0.66.

Many genetic variants are unclassified due to insufficient evidence. For these, we estimated how many cases would be needed to resolve this unclassified status using a simulation study based on the multifactorial likelihood model. If a *BRCA1* variant was genuinely benign but was unclassified at the time of analysis, only one case with this same variant would be needed to be classified as (likely) benign with a 0.049 or less posterior probability at a prior probability of 0.02–0.03, 5 cases at 0.26–0.29, 7 cases at 0.66, 10 cases at 0.81, and 12 cases at 0.96 (Figure 2a, Table S8). If an unclassified *BRCA1* variant was genuinely pathogenic, 9 cases with this variant would be required to reach (likely) pathogenic class (with 0.95 or more posterior probability) at a prior probability 0.02–0.03, 7 cases at 0.26, 6 cases at 0.29, 4 cases at 0.66, 3 cases at 0.81, and 1 case at 0.96 (Figure 2b, Table S8).

If an unclassified *BRCA2* variant was really benign, 1 case at a prior probability of 0.02–0.03, 20 cases at 0.26, 21 cases at 0.29, 31 cases at 0.66, 36 cases at 0.81, and 44 cases at 0.96 would be needed to downgrade this to a (likely) benign variant (Figure 2c, Table S9). If the *BRCA2* VUS was actually pathogenic, 28 cases at a prior probability of 0.02–0.03, 20 cases at 0.26, 19 cases at 0.29, 12 cases at 0.66, 9 cases at 0.81, and only 1 case at 0.96 would be needed to upgrade this variant to a (likely) pathogenic variant (Figure 2d, Table S9).

We then estimated how much the posterior probability is affected by whether or not clinical information such as pathologic profiles or personal/family cancer history exists, with specific focus on BRCA2, c.7522G>A, p.(Gly2508Ser) (Figure 2e, Table S10) and BRCA1, c.5339T>C, p.(Leu1780Pro) (Figure 2f, Supplementary Table S11). The prior probability of these two variants was the same at 0.66, and these were both classified as VUS in our previous study [18]. However, by registering more cases with these two variants in the current study, the BRCA2, c.7522G>A, p.(Gly2508Ser) variant was downgraded from IARC 3 (total 1 case, posterior probability 0.562) to IARC 2 (total 11 cases, posterior probability 0.047), while the BRCA1, c.5339T>C, p.(Leu1780Pro) variant was upgraded from IARC 3 (total 4 cases, posterior probability 0.652) to IARC 5 (total 26 cases, posterior probability 1).

In the case of the *BRCA2*, c.7522G>A, p.(Gly2508Ser) variant, using simulation analysis, if the missing rate was 0%, the posterior probability in 1 case was estimated to be 0.577, and posterior probability went down to 0.135 when the total number of samples reached 11. However, if missing rate was up to 75%, the posterior probability was 0.659 in 1 case, and even when 11 cases were accumulated, the posterior probability was barely lowered (0.640). In the case of *BRCA1* c.5339T>C, p.(Leu1780Pro), if the missing rate was 0%, the posterior probability in one case was 0.764, and it went up to 0.999 when there were 26 sample cases; however, if the missing rate was up to 75%, the posterior probability in 26 cases was predicted to be 0.952. With this simulation study of the *BRCA1* c.5339T>C variant, we could estimate the number of cases required to upgrade from IARC 3 to IARC 4 depending on the missing rate. If the missing rate was 0%, 7 cases (posterior probability 0.959) were required, while 9 cases were required at a missing rate of 25%, 13 cases were required at a missing rate of 50%, and 26 cases were required at a missing rate of 75%.

### 2.4. Analysis of BRCA1/2 PLPVs in the Korean BRCA

In the Korean general population in gnomAD (non-cancer, v.2.1.1, 1887 subjects), 5 different *BRCA1* PLPVs in 7 carriers and 6 different *BRCA2* PLPVs in 7 carriers were found. Finally, there were 130 different *BRCA1* PLPVs and 112 *BRCA2* PLPVs in the Korean BRCA. In *BRCA1*, c.81-9C>G, c.390C>A (p.(Tyr130*)), c.2433del (p.(Lys812Argfs*3)), c.3627dup (p.(Glu1210Argfs*9)), c.5339T>C (p.(Leu1780Pro)), and c.5445G>A (p.(Trp1815*)) were found only in the Korean population in gnomAD (v.2.1.1), while c.1399A>T (p.(Lys467*)) and c.3744_3747del (p.(Ser1248Argfs*10)) in *BRCA2* were found only in the Korean and not in other populations in gnomAD.

**Figure 3:**
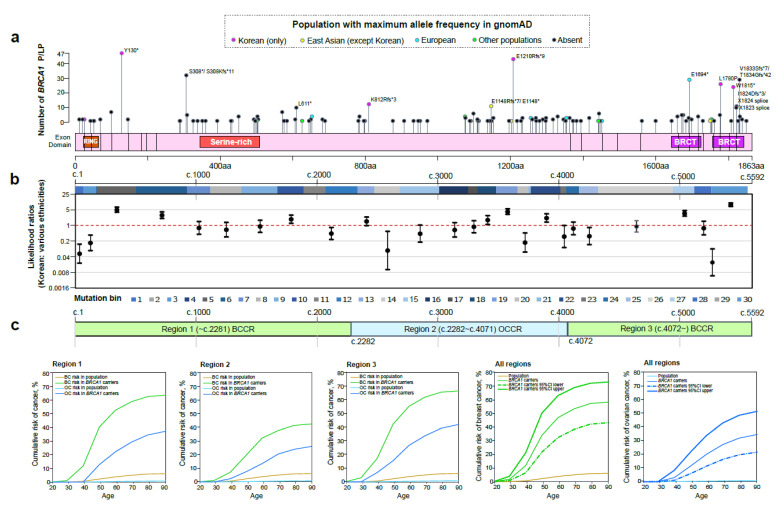
Analysis of pathogenic or likely pathogenic variants in *BRCA1.* (**a**) Distribution of pathogenic or likely pathogenic variants (PLPVs) in *BRCA1* in the Korean BRCA cohort. The population in gnomAD with maximum allele frequency at individual PLPVs is marked in each color in the circle of lollipop. (**b**) Comparative analysis of the proportion of *BRCA1* PLPVs found in every mutation bin between the Korean BRCA and a large-scale cohort including various ethnicities over 6 continents. The 30 mutation bins across the span of the coding DNA sequence of *BRCA1* were constructed using an algorithm in which each mutation bin contained approximately equal numbers of *BRCA1* PLPVs in a previous study [21]. (**c**) Estimation of cumulative risks of breast and ovarian cancers for *BRCA1* carriers by mutation region: region 1 (5′ to c.2281, breast cancer cluster regions (BCCR)), region 2 (c.2282 to c.4071, ovarian cancer cluster regions (OCCR)), and region 3 (c.4072 to 3′, BCCR) [21,22]. RING, RING finger domain (24–64) by Pfam (http://pfam.xfam.org/ (accessed on 1 March 2021)); Serine-rich, serine-rich domain associated with BRCT (344–508); BRCT, BRCA1 C Terminus domain (1662–1723, 1756–1842); P/LP, pathogenic or likely pathogenic variants; BC, breast cancer; OC, ovarian cancer.

**Figure 4:**
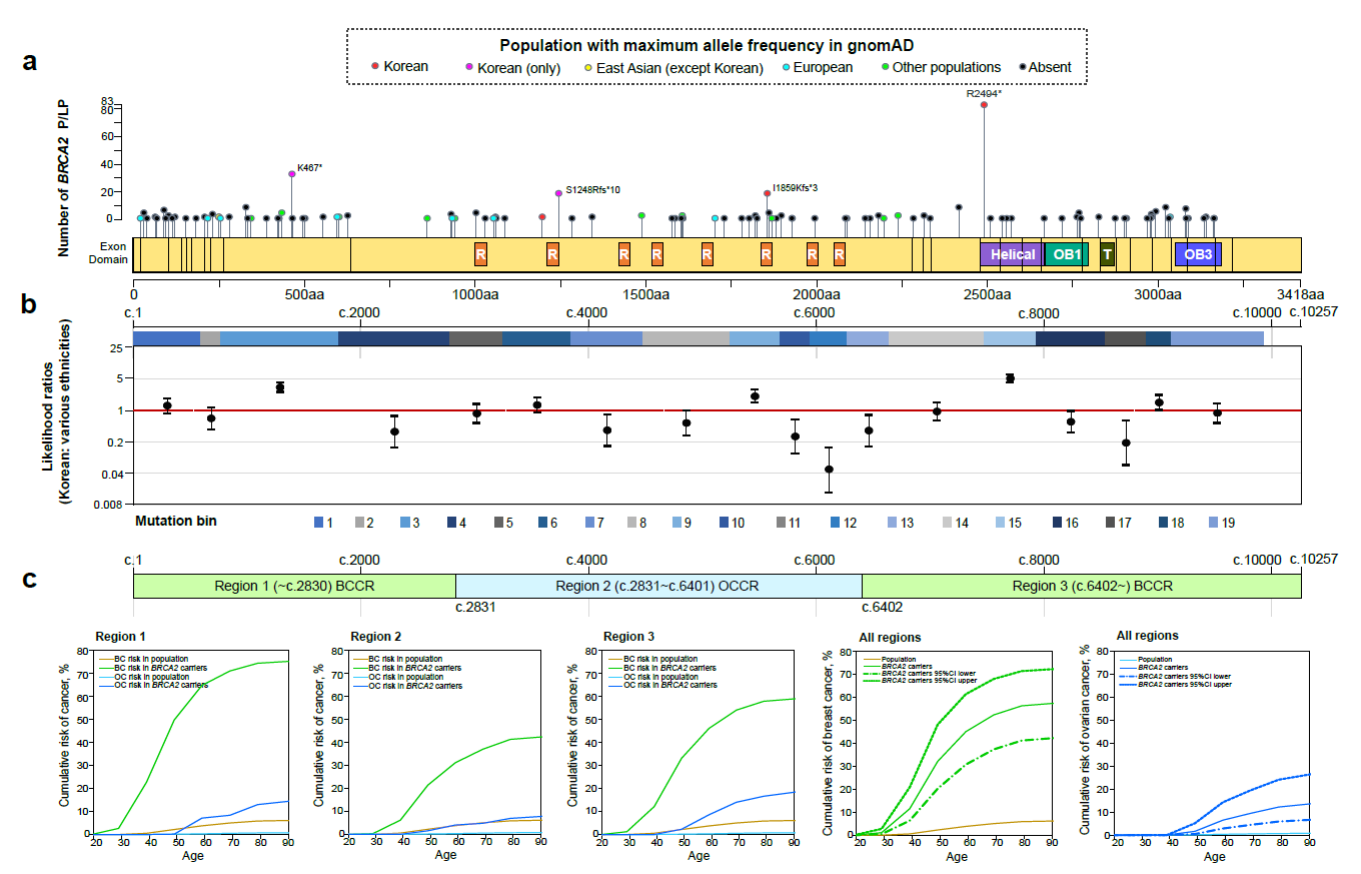
Analysis of pathogenic or likely pathogenic variants in *BRCA2*. (**a**) Distribution of pathogenic or likely pathogenic variants (PLPVs) in *BRCA2* in the Korean BRCA cohort. (**b**) Comparative analysis of the proportion of *BRCA2* PLPVs found in every mutation bin between the Korean BRCA and large-scale cohort. The 19 mutation bins across the span of the coding DNA sequence of *BRCA2* were constructed using an algorithm in which each mutation bin contained approximately equal numbers of *BRCA2* PLPVs in a previous study [21]. (**c**) Estimation of cumulative risks of breast and ovarian cancers for *BRCA2* carriers by mutation regions: region 1 (5′ to c.2830, breast cancer cluster regions (BCCR)), region 2 (c.2831 to c.6401, ovarian cancer cluster regions (OCCR)), and region 3 (c.6402 to 3′, BCCR) [21,22]. R, BRCA2 repeat (1002–1035, 1213–1245, 1422–1454, 1518–1550, 1665–1696, 1838–1869, 1973–2004, 2052–2084) by Pfam (http://pfam.xfam.org/ (accessed on 1 March 2021)); Helical, BRCA2 helical (2481–2667); OB1, oligonucleotide/oligosaccharide-binding, domain 1 (2670–2796); T, Tower (2831–2872); OB3, oligonucleotide/oligosaccharide-binding, domain 3 (3052–3186); P/LP, pathogenic or likely pathogenic variants; BC, breast cancer; OC, ovarian cancer.

We searched for the population in the gnomAD that showed the maximum allele frequency at individual PLPVs found in the Korean BRCA. Most *BRCA1* (83.1%, 108/130) or *BRCA2* (77.7%, 87/112) PLPVs found in the Korean BRCA were absent in gnomAD, followed by Korean (4.6%, 6/130), European (Finnish or non-Finnish) (3.8%, 5/130) or other populations (South Asian, Latino, or other, 3.8%, 5/130), and East Asian (except Korean, 2.3%, 3/130) in *BRCA1* (Figure 3a), while followed by other populations (African/African American, Latino, Ashkenazi Jewish, or South Asian, 9.8%, 11/112), European (7.1%, 8/112), Korean (4.5%, 5/112), and East Asian (0.9%, 1/112) in *BRCA2* (Figure 4a).

Compared with the proportion of *BRCA1*/*2* PLPVs in a large-scale cohort including various ethnicities [21], the *BRCA1* PLPVs in mutation bins 5, 6, 10, 18, 19, 22, 27, and 30 (Figure 3b) and the *BRCA2* PLPVs in mutation bins 3, 9, 15, and 18 (Figure 4b) found in the Korean BRCA showed statistically increased likelihood ratios (Table S12).

### 2.5. Estimating the Risks of Breast and Ovarian Cancers in BRCA1 and BRCA2 Carriers

Our estimates of breast and ovarian cancer risk for *BRCA1* or *BRCA2* carriers are shown in Figure 3c and Figure 4c, respectively. The cumulative lifetime risk of breast cancer for *BRCA1* carriers was 59.1% (95% CI: 44.1–73.6%): 64.4% (95% CI: 37.8–87.1%) in region 1 (~c.2281, BCCR), 43.4% (95% CI: 22.4–69.8%) in region 2 (c.2282 to c.4071, OCCR), and 67.3% (95% CI: 41.1–88.3%) in region 3 (c.4072~, BCCR) (Figure 3c, Supplementary Table S13). The lifetime cumulative risk of ovarian cancer for *BRCA1* carriers was estimated to be 36.9% (95% CI: 23.4–53.9%): 39.7% (95% CI: 16.6–72.8%) in region 1, 28.1% (95% CI: 12.1–55.8%) in region 2, and 44.4% (95% CI: 19.9–76.0%) in region 3 (Figure 3c, Supplementary Table S13). The lifetime cumulative risk of breast cancer for *BRCA2* carriers was similar to that of *BRCA1*. It was 58.3% (95% CI: 43.2–73.0%), and there were also differences in the risk by region: 76.1% (95% CI: 42.8–96.0%) in region 1 (~c.2830, BCCR), 43.4% (95% CI: 22.4–69.8%) in region 2 (c.2831 to c.6401, OCCR), and 60.0% (95% CI: 37.5–81.1%) in region 3 (c.6402~, BCCR) (Figure 4c, Supplementary Table S14). The lifetime cumulative risk of ovarian cancer for *BRCA2* carriers was 14.9% (95% CI: 7.4–28.5%): 15.8% (95% CI: 2.4–70.7%) in region 1, 8.6% (95% CI: 2.3–28.6%) in region 2, and 20.0% (95% CI: 7.5–46.4%) in region 3 (Figure 4c, Supplementary Table S14).

## 3. Discussion

In the present study, our major question was how causative *BRCA1* or *BRCA2* variants in non-European populations differ from those in European populations, which are most studied. Another question was how different were the clinical outcomes caused by their variants. We considered *BRCA1/2* VUS and PLPVs in this study.

The *BRCA1/2* VUS has been investigated in the European population with large-scale research based on clinical information associated with *BRCA1/2* [8,23,24,25]. These efforts have made it possible for recurrent VUS detected in European population to be reclassified as pathogenic or benign variants. In particular, an IARC classification for *BRCA1* and *BRCA2* using a multifactorial likelihood model has made a great contribution to reclassifying VUS into (likely) benign or (likely) pathogenic variants. Recently, a large-scale multifactorial likelihood analysis for *BRCA1* and *BRCA2* was applied by Evidence-based Network for the Interpretation of Germline Mutant Alleles (ENIGMA) [8]. When this multifactorial probability-based model is applied, integral analyses such as personal or family history, pathologic profiles, or segregation LRs are needed. However, to date, these LRs are estimated based on the clinical characteristics of the European population. These are limitations to applying such LRs in non-European populations. To overcome these limitations, although there was a limit to the study of small numbers, we previously estimated the LRs of personal and family history, pathologic profiles, and co-occurrence with pathogenic variants in Korean patients with breast cancer [18].

In this study, data from our previous study was extended and also included Korean ovarian cancer. Interestingly, we found that some of the *BRCA1* or *BRCA2* VUS reported in the previous study changed their variant classes to (likely) benign variants, with two gold stars of review status in ClinVar (accessed on 17 March 2020). In the two cases (c.7522G>A, p.(Gly2508Ser) in *BRCA2* and 5339T>C, p.(Leu1780Pro) in *BRCA1*) where the prior probability (0.66) was the same and posterior probability was similar in the previous study, posterior probability and their classification have completely changed as clinical cases with these variants accumulated. The c.7522G>A, p.(Gly2508Ser) in *BRCA2* went from IARC 3 (total 1 case, posterior probability 0.562) to IARC 2 (total 11 cases, posterior probability 0.047), and c.5339T>C, p.(Leu1780Pro) in *BRCA1* went from IARC 3 (total 4 cases, posterior probability 0.652) to IARC 5 (total 26 cases, posterior probability 1). After our study, the 5339T>C, p.(Leu1780Pro) in *BRCA1* was revealed as a Korean founder pathogenic variant [26,27].

In particular, clinical genetic laboratories should provide genetic test reports by conducting clinical interpretations of genetic variants with genetic testing within a specified turnaround time. Therefore, whenever a very rare variant is detected, there is a limit to conducting a functional study for it. In this respect, the variant classification using a multifactorial likelihood model that does not require functional research can be useful. The KONCORD consortium is a network consisting of clinical genetic laboratories that, when a rare variant is detected, is configured to quickly collect cases with the same corresponding variant detected, using the network to perform an integrated analysis. Therefore, to take full advantage of this network, it is important to predict how many samples can solve the unclassified status when collected, along with to recognize the limitations of using the multifactorial likelihood model.

For these, we applied a simulation analysis for *BRCA1/2* VUS. The smaller the number of samples, the more the posterior probability is affected by prior probability, the more samples for *BRCA2* VUS are needed to resolve unclassified status compared with *BRCA1* VUS, and the presence of clinical information associated with VUS is an important factor. First, prior probability is important when there are a small number of samples. For example, the posterior probability of c.5017_5019del, p.(His1673del) in *BRCA1* (total 5 cases, combined LR 146.848019) in our study was 0.999716 (class 5, pathogenic), while the posterior probability of this variant (total 9 cases, combined LR 29.735396) in the ENIGMA study [8] was estimated to be 0.479072 (class 3, uncertain significance). The most important cause of this difference was a different prior probability applied in these two studies: we applied a 0.96 prior probability for this variant using the VEST-indel algorithm (CRVAT), while a 0.03 prior probability was applied in the ENIGMA study because Align-GVGD does not score in-frame indels; therefore, the highest Align-GVGD priority of the deleted based was applied. The predictions of pathogenicity for indel variants are not as well established as those for single nucleotide variants. We checked the prediction results of pathogenicity for this variant using other in silico tools: damaging with confidence score 0.894 by SIFT indel, neutral with −2.168 score by PROVEAN. In the previous study, the accuracy of any in silico prediction for indels did not reach >90% [28]. If the prior probability of c.5017_5019del, p.(His1673del) in *BRCA1* in this study is greater than 0.115, this variant could be classified as (likely) pathogenic (posterior probability > 0.95) because the combined LR is 146.848019. In addition, there were several VUS cases where the value of the prior probability was not consistent with the functional impact result by known functional assays: c.5131A>G (p.Lys1711Glu), c.5137G>T (p.Val1713Leu), c.5254G>A (p.Ala1752Thr), and c.5365G>C (p.Ala1789Pro) in *BRCA1*; c.8702G>A (p.Gly2901Asp), c.9275A>G (p.Tyr3092Cys), and c.9367A>G (p.Ser3123Gly) in *BRCA2* (Supplementary Tables S6 and S7). Therefore, there is a limit to the accuracy of the prior probability; so, if the number of samples is small, care should be taken when analyzing the variants using the multifactorial likelihood model.

With simulation study, we could predict that an additional minimum of 12 cases with the same variants in *BRCA1* would be needed to classify these as (likely) benign without relying on the prior probability; an additional minimum of 9 clinical cases with same variants in *BRCA1* would be required to reclassify variants as (likely) pathogenic. An additional minimum of 44 clinical cases with the same variants in *BRCA2* would be required to reclassify variants as (likely) benign, and 28 cases would be required to reclassify variants as (likely) pathogenic in *BRCA2*. To resolve unclassified status, more samples are needed for *BRCA2* VUS than *BRCA1* VUS. This is caused by a difference in the range of estimated combined LRs of real pathogenic variants between *BRCA1* and *BRCA2*. In addition, the combined LRs of all VUS in *BRCA1* were <0.5 or >2, while only 44.8% (47/105) VUS in *BRCA2* had an LR within that range in this study. The lack of clinical information associated with related VUS might lead to a greater difference in the posterior probability results of VUS between *BRCA1* and *BRCA2*. For better resolution of this indeterminate status in *BRCA2*, other clinical factors that highlight the differentiation between pathogenic and neutral status should be included in the multifactorial likelihood analysis.

Based on current cancer statistics in the USA (https://seer.cancer.gov/csr/1975_2017/ (accessed on 5 February 2021), including multiple ethnicities), 12.9% of women will develop breast cancer and 1.2% of women will develop ovarian cancer during their lifetime. In Korea, the breast and ovarian cancer risk in the general population is 6.4% and 0.97%, respectively, based on our analysis derived from the annual report of cancer statistics in Korea (https://ncc.re.kr/main.ncc?uri=english/sub04_Statistics (accessed on 15 October 2020) (Tables S13 and S14). The previous study based on the data from the Consortium of Investigators of Modifiers of BRCA1/2 (CIMBA) has identified that there was a substantial difference in variant type, frequency, and location by geographical region and race or ethnicity [10]. In addition, over 50% of Asian *BRCA* variants were Asian-specific, and even among Asian populations (Indian, Chinese, Korean, and Japanese populations) there were significant differences [11]. For these reasons, we assumed that the patterns and effects of *BRCA1* or *BRCA2* pathogenic variants related to breast or ovarian cancer in Korean are different than those of other populations (not only non-Europeans but also other Asians).

In the current study, we found that there were regions of pathogenic variants in *BRCA1* or *BRCA2* specially enriched in the Korean population (the *BRCA1* PLPVs in mutation bins 5, 6, 10, 18, 19, 22, 27, and 30 and the *BRCA2* PLPVs in mutation bins 3, 9, 15, and 18). Although there was a higher risk of breast or ovarian cancer in other general populations compared with the Korean general population (in particular, the other general populations have double the risk of breast cancer), the cumulative risks of breast or ovarian cancer for Korean *BRCA1* or *BRCA2* carriers were comparable with those of European carriers [22,29,30,31,32,33,34,35,36] (Tables S15 and S16). Therefore, in the population-based studies, the odds ratios for breast cancer are higher in Asian than in European populations when comparing case patients with controls: odds ratio 9.33 (95% CI: 7.00–12.43) for *BRCA1* (protein-truncating variants) in Europeans, odds ratio 22.07 (95% CI: 6.91–70.48) for *BRCA1* in Asians, odds ratio 5.38 (95% CI: 4.38–6.59) for *BRCA2* in Europeans, and odds ratio 8.16 (95% CI: 4.90–13.57) for *BRCA2* in Asians in the Breast Cancer Association Consortium’s study [31]; odds ratio 29.06 (95% CI: 11.98–70.51) for *BRCA1* (protein-truncating variants) in Koreans, odds ratio 23.77 (95% CI: 10.57–53.50) for *BRCA2* in Koreans in this study (Table S15). Interestingly, the cumulative risks of breast or ovarian cancer for Korean *BRCA1* or *BRCA2* carriers by region of BCCR or OCCR showed a different trend than what was previously reported [22] (Tables S15 and S16). The cumulative risks for both breast and ovarian cancer at OCCR were not higher than those at the other two BCCRs for both Korean *BRCA1* and *BRCA2* carriers. However, since the number of samples in this study was not as large as in the previous study [22], further research is required to verify them.

## 4. Materials and Methods

### 4.1. Participants

All centers of the KONCORD consortium obtained written informed consent from participants, and local institutional review boards approved this study protocol. Study eligibility criteria included Korean subjects (aged ≥18 years) who underwent whole *BRCA1* or *BRCA2* gene testing due to suspicion of high risk of familial breast–ovarian cancer. Clinical data including sex, age, cancer site, onset age, family history of cancer, and laboratory data including *BRCA1/2* genetic testing results and histologic diagnosis (including immunohistochemical stain results) were collected in this study.

### 4.2. Initial Classification of BRCA1 and BRCA2 Variants

Initially, we classified pathogenic, likely pathogenic, likely benign, and benign variants considering 2015 ACMG/AMP criteria [3] or variant classification with review status in ClinVar (https://www.ncbi.nlm.nih.gov/clinvar/, accessed on 17 March 2020). The *BRCA1* and *BRCA2* variants were analyzed based on NM_007294.3 (between c.1 and c.5563 region) and NM_000059.3 (between c.1 and c.9925), respectively. Loss-of-function variants in *BRCA1* or *BRCA2* genes are responsible for the development of a hereditary breast–ovarian cancer syndrome [37]. Therefore, the pathogenic or likely pathogenic variants (PLPVs) included null variants such as stop-gain, splice site disrupting, frameshift, or initiation codon variants and other PLPVs according to the 2015 ACMG/AMP criteria. Variants with ≥5% allele frequency (BA1) in gnomAD (https://gnomad.broadinstitute.org/ (accessed on 13 March 2020)) and other (likely) benign variants according to 2015 ACMG/AMP were classified as (likely) benign variants. The remainder, including VUS with ≥two gold stars of review status in ClinVar, or any variants with <two gold stars of review status in ClinVar, were classified as VUS.

### 4.3. IARC Classification of Variants of Uncertain Significance

The IARC classification of VUS (which is the multifactorial probability of a VUS being pathogenic) is based on Bayes theorem using the prior probability of causality and combined likelihood ratio [24]. For the combined LR, we created a benign and pathogenic (BRCA1/2+) group. The benign group included individuals with only (likely) benign variants in *BRCA1* or *BRCA2*, while the pathogenic group included *BRCA1* PLPV carriers (BRCA1+) and *BRCA2* PLPV carriers (BRCA2+).

We considered LRs for personal/family cancer history, pathologic profiles, and co-occurrence with pathogenic variants between benign and pathogenic groups following our previous study [18]. Briefly, 20 categories were used in the interval LR of personal/family cancer history as follows: cancer type (unilateral breast cancer, bilateral breast cancer, breast and ovarian cancer, ovarian cancer), age at diagnosis of cancer (<40 or ≥40 years in unilateral breast cancer, <45 or ≥45 years in bilateral breast cancer), and number of family members with breast, ovarian, prostate, or pancreatic cancer (0, 1, 2, and ≥3 in unilateral breast cancer; 0, ≥1 in bilateral breast cancer, breast and ovarian cancer, or ovarian cancer). For the likelihood of pathologic profile, the breast cancers were categorized into luminal, HER2, and basal-like type [38], combined with histologic grade (Bloom–Richardson grade), and ovarian cancers were classified into two groups as carcinoma in situ/low grade (grade 1) and high grade (grades 2 and 3). The LR of co-occurrence was analyzed using an LR formulation introduced by the previous study [23]. To determine the prior probability, we used in silico algorithms for missense or splicing variants using PRIORS (http://hci-priors.hci.utah.edu/PRIORS/index.php (accessed on 23 March 2020)) [19] and indel variants using CRAVAT (http://cravat.us/CRAVAT/ (accessed on 23 March 2020)) [39], which included the variant effect scoring tool (VEST) indel algorithm [28].

### 4.4. Simulation Study Estimating the Number of Additional Samples Required to Resolve Unclassified Status

We estimated how many samples were needed to reach ≥IARC 4 or ≤IARC 2 when a *BRCA1* or *BRCA2* genetic variant was unclassified. For this, we created two benign and pathogenic datasets, independent from the benign and pathogenic model groups. Following recommendations by the previous study [20], a posterior probability of 0.049 or less (IARC 2 or less) was regarded as a (likely) benign class, while a posterior probability of 0.95 or more (IARC 4 or more) was classified as a (likely) pathogenic class. The benign set contained samples that had only (likely) benign variants, while the pathologic set contained samples that had (likely) pathogenic variants in *BRCA1/2*.

Then, for each benign and pathologic set, we sampled LR scores of pathologic profiles and personal/family cancer history independent from each other with replacement and calculated the posterior probability with the assumption that there was no co-occurrence with other pathologic variants. The calculation was repeated 1000 times for the different prior probabilities [24] against increasing the number of patients, *N* (Supplementary Tables S8–S11). Figure 2. shows the mean posterior probability of 1000 calculations against the number of patients.

### 4.5. Analysis of BRCA1/2 PLPVs in the Korean BRCA

The *BRCA1/2* PLPVs ((likely) pathogenic variants by 2015 ACMG/AMP or IARC 4/5 variants by prior our cohort analysis) were identified in gnomAD Korean (non-cancer, v.2.1.1). We combined the data on *BRCA1/2* PLPVs from our cohort and the Korean general population (in gnomAD), and this was then referred to as the Korean BRCA. The proportion of *BRCA1/2* PLPVs found in every mutation bin was compared between the Korean BRCA and a previous large-scale cohort (including various ethnicities in six continents) [21] using interval likelihood ratios. Mutation bins across the span of the coding DNA sequence of *BRCA1* (30 mutation bins) or *BRCA2* gene (19 mutation bins) were defined by a previous study [21]. They were constructed using an algorithm in which each mutation bin contained near-equal numbers of PLPVs, with bin length defined by distance in base pairs. Mutationmapper (https://www.cbioportal.org/mutation_mapper (accessed on 24 October 2020)) was used and modified to simultaneously visualize genetic lesions.

### 4.6. Estimation of Risks of Breast and Ovarian Cancers for BRCA1 and BRCA2 Carriers

A Bayesian calculation was applied to determine the risks of breast or ovarian cancer in *BRCA1* or *BRCA2* PLPV carriers using the following formula [40,41]:

$$P(D|G)=\frac{P(G|D)P\left(D\right)}{P(G|D)P\left(D\right)+P(G|\bar{D})(1-P(D))}$$

In this formula, *D* = disease, *G* = genotype, $\bar{D}$ = absence of disease, *P*(*D*|*G*) = penetrance, *P*(*G*|*D*) = genotype frequency in cases, $P(G|\bar{D})$ = genotype frequency in controls, and *P*(*D*) = general population prevalence for breast or ovarian cancers. We defined the frequency of disease (*P*(*D*)) as the cumulative risk of breast or ovarian cancers by age in the Korean general population, based on analysis of the data from the annual report of cancer statistics in Korea in 2016 (https://ncc.re.kr/main.ncc?uri=english/sub04_Statistics (accessed on 15 October 2020)). For the genotype frequency in controls ($P(G|\bar{D})$), we used the *BRCA1/2* PLPVs ((likely) pathogenic variants by 2015 ACMG/AMP or IARC 4/5 variants identified by prior our cohort analysis) in gnomAD Korean (non-cancer, v.2.1.1). Due to insufficient epidemiologic information (e.g., age, sex) of participants in gnomAD, we could not consider differences in *P*(*G*|$\bar{D}$) by age or sex. The 95% confidence interval (CI) for penetrance was calculated using binomial probability [40,41]. We considered the risks of breast or ovarian cancer for *BRCA1*/*2* carriers by mutation regions. For *BRCA1*, 5′ to c.2281 (region 1: breast cancer cluster regions, BCCR), c.2282 to c.4071 (region 2: ovarian cancer cluster regions, OCCR), and c.4072 to 3′ (region 3: BCCR) were considered, while *BRCA2* considered 5′ to c.2830 (region 1: BCCR), c.2831 to c.6401 (region 2: OCCR), and c.6402 to 3′ (region 3: BCCR) [21,22].

## 5. Conclusions

To date, large-scale studies related to *BRCA1* or *BRCA2* have focused on European populations. Although we had limited data, we tried to identify the difference in the spectrum of *BRCA1* or *BRCA2* variants and the related clinical outcomes between other populations (mainly Europeans) and Koreans from various perspectives. Data from this study reveal that *BRCA 1* or *BRCA2* variants in the non-European population is highly specific; therefore, population-specific study is essential for clinical application of treatment or prevention for breast or ovarian cancer.

## Data Availability

All data analyzed in this study are included in this article and its supplementary information files.

## Supplementary Materials

The following are available online at https://www.mdpi.com/article/10.3390/cancers13092192/s1, Table S1: Demographics of study participants, Table S2: The pathologic subtypes of breast or ovarian cancer enrolled, Table S3: BRCA1 (likely) pathogenic variants in our cohort according to the 2015 ACMG/AMP standards and guidelines, Table S4: BRCA2 (likely) pathogenic variants in our cohort according to the 2015 ACMG/AMP standards and guidelines, Table S5: Likelihood ratios of personal and family history compared BRCA1/2 (likely) pathogenic variants carriers to noncarriers, Table S6: IARC classification of BRCA1 variants of uncertain significance using multifactorial posterior probability analyses, Table S7: IARC classification of BRCA2 variants of uncertain significance using multifactorial posterior probability analyses, Table S8: Prediction of posterior probability of BRCA1 variants depending on the prior probability using random sampling, Table S9: Prediction of posterior probability of BRCA2 variants depending on the prior probability using random sampling, Table S10: Prediction of posterior probability of c.7522G>A, p.(Gly2508Ser) in BRCA2 depending on the presence of clinical information using random sampling, Table S11: Prediction of posterior probability of c.5339T>C, p.(Leu1780Pro) in BRCA1 depending on the presence of clinical information using random sampling, Table S12: Comparative analysis of the proportion of number of BRCA1/2 (likely) pathogenic variants found at every mutation bin between the Korean BRCA and large-scale cohort, Table S13: Estimation of risks of breast and ovarian cancers for BRCA1 carriers, Table S14: Estimation of risks of breast and ovarian cancers for BRCA2 carriers, Table S15: Previous reports on breast cancer risk for BRCA1 and BRCA2 carriers, Table S16: Previous reports on ovarian cancer risk for BRCA1 and BRCA2 carriers.
